# Is monkeypox an STI? The societal aspects and healthcare implications of a key question

**DOI:** 10.12688/wellcomeopenres.18436.1

**Published:** 2022-10-11

**Authors:** Jaime Garcia Iglesias, Maurice Nagington, Martyn Pickersgill, Michael Brady, Claire Dewsnap, Liz Highleyman, Francisco Javier Membrillo de Novales, Will Nutland, Steven Thrasher, Eric Umar, Ian Muchamore, Jamie Webb

**Affiliations:** 1 Centre for Biomedicine, Self and Society, University of Edinburgh, Edinburgh, UK; 2 School of Health Sciences, University of Manchester, Manchester, UK; 3 King's College Hospital NHS Foundation Trust, London, UK; 4 Sexual Health, Sheffield Teaching Hospital NHS Foundation Trust, Sheffield, UK; 5 POZ Magazine, New York City, USA; 6 CBRN and Infectious Diseases Unit, Hospital Central de la Defensa "Gomez Ulla", Madrid, Spain; 7 The Love Tank CIC, London, UK; 8 Medill School of Journalism, Media, Integrated Marketing Communications, Northwestern University, Evanston, USA; 9 Kamuzu University of Health Sciences, Blantyre, Malawi; 10 Centre for Technomoral Futures, University of Edinburgh, Edinburgh, UK

**Keywords:** Monkeypox, STI, sex, sexual health, public health, social sciences

## Abstract

This letter explores the societal aspects and healthcare implications that underlie thinking about monkeypox, in the 2022 outbreak, as a sexually transmitted infection (STI). The authors examine what underlies this question, exploring what is an STI, what is sex, and what is the role of stigma in sexual health promotion. The authors argue that, in this specific outbreak, monkeypox is an STI among men who have sex with men (MSM). The authors highlight the need of critically thinking about how to communicate effectively, the role of homophobia and other inequalities, and the importance of the social sciences.

## Disclaimer

The views expressed in this article are those of the author(s). Publication in Wellcome Open Research does not imply endorsement by Wellcome.

## Introduction

Since May 2022, non-endemic countries have been experiencing an outbreak of monkeypox. On July 23 ^rd^, 2022, the World Health Organization (WHO) declared monkeypox “a public health emergency of international concern” and, in August, the White House declared it a “public health emergency.” At the time of writing (September 30, 2022), over 67,000 confirmed cases have been reported across 106 countries, mostly in Europe and the Americas by WHO.

Compared with previous or historic outbreaks in Africa (and, particularly, in Nigeria), the current outbreak presents some significant differences: over 97% of reported cases are male; among cases with available sexual orientation data, over 89% are gay, bisexual, or other men who have sex with men (MSM); a sexual encounter is the most commonly reported type of transmission (>87%) and a ‘party with sexual contacts’ the most likely reported exposure setting (over 50%). Case manifestations also differ, with anogenital lesions and single lesions being more common than in previous outbreaks ( Català *et al.*, 2022; Thornhill *et al.*, 2022). These differences have led many ( Fischer 2022, Highleyman 2022, Moniuszko 2022) to wonder: Is monkeypox, in the 2022 outbreak, a sexually transmitted infection (STI)?

Characterizing monkeypox as an STI does nothing to alter the biological realities of the virus, its symptoms, or the pain afflicted people experience. The wider implications, however, are numerous. At the individual and practical level, it might help stimulate the development of robust and targeted information about transmission for those most at risk. At the public health system and health service delivery levels, it will shape and influence key decisions around the surveillance and management of this current outbreak. At the policy and economic level, it will release - or in some cases limit - funding and political urgency. At the conceptual level, it will involve debates about the meanings of health, disease, and sex. Here, we examine what underlies this larger question, exploring what is an STI, what is sex, and what is the role of homophobia in sexual health promotion. We argue that, in this specific outbreak, monkeypox is, in effect, an STI among MSM. This, in turn, underscores the need for important conversations about how to communicate effectively, the role of homophobia and other inequalities, and the importance of the social sciences and societal transformations.

## What is an STI?

The World Health Organization defines sexually transmitted infections as those which are “transmitted through sexual contact, including vaginal, anal and oral sex.” Classic examples of STIs include syphilis, chlamydia, HIV, and gonorrhea. This definition, however, is somewhat more complicated on two fronts. First, some of the better known STIs, such as HIV, are also frequently transmitted non-sexually. For example, many countries have experienced outbreaks of HIV among people who inject drugs (e.g. Paraskevis *et al.*, 2013). Conversely, some diseases that are not widely recognized as STIs, such as hepatitis C, can be transmitted via sex. Second, speaking about sexual transmission requires some shared understanding of what we consider to be ‘sex’. As we will see in the next section, this is not necessarily straightforward.

Defining monkeypox as an STI would imply that the responsibility for managing it falls to sexual health services, where they exist. There are clear benefits to this: Sexual health clinicians and community partners have a wealth of expertise in developing effective interventions and messages that target men who have sex with men and other groups at risk for STIs, in the face of stigma ( Race, 2021). However, sexual health is often chronically underfunded and tends to be difficult to access ( Iacobucci & Torjesen, 2017). Adding acute or ongoing monkeypox outbreaks to the workload of sexual health providers, without building additional capacity, will deepen existing inequalities and access problems.

Defining monkeypox as an STI may also transform how it is perceived. Far from the global, societal threat that characterizes COVID-19, considering monkeypox to be an STI may cause it to be perceived as a problem only for certain individuals. In the case of HIV, the advent of effective medications in the Global North contributed to policy shifting from seeing the virus as a societal issue to seeing it as an individual health condition, thwarting social action and deepening inequalities ( Catalan *et al.*, 2021; Kagan, 2018).

## What is sex?

In recent decades, there has been a broadening and transformation of the range of practices generally considered to be sexual contact. This includes the development of new technologies and the incorporation into the mainstream of traditionally minority activities, such as kink or BDSM ( Plummer, 2003; Sundén & Paasonen, 2020; Wignall, 2022). For example, a recent debate in the *BMJ* centered on whether women engaging in ‘anal sex’ had specific sexual health needs ( Gana & Hunt, 2022, see rapid responses). People who engage in more novel or previously less visible (to the mainstream) practices require targeted sexual health promotion and care, both because of the practical implications of some of those practices and because of the oftentimes negative societal perceptions and stigma that surround them ( McGregor, 2015; Waldura *et al.*, 2016; Sprott & Randall, 2017).

Different sexual practices may be related to diverse clinical presentations ( Tarín-Vicente *et al.*, 2022). The range of practices that specific communities, such as MSM, associate with sex but which do not consist of penile penetration also needs to be taken into account in the development of health promotion around monkeypox. Community organizations have, for instance, identified the need to develop guidance that directly addresses particular sex practices such as the eroticized wearing of rubber or leather, bondage, or watersports. These practices, however, may not always be recorded as ‘sex’ in surveys or statistical data, demonstrating how slippery the notion of ‘sex’ can be.

## What is the role of stigma in sexual health promotion?

If monkeypox is defined as an STI, it will be directly associated with sex and, more specifically, with ‘gay sex,’ since men who have sex with men – often wrongly subsumed under the label ‘gay’ – remain disproportionally affected in the current outbreak. Commentators have argued that this might lead to deepening stigmatization and further attacks on LGBT people, who might be seen as ‘dirty’ or ‘reckless,’ and it could also become a tool to further criminalize sex between men. This is far from new: HIV has, for decades, been leveraged to legitimize and justify pre-existing homophobic, transphobic, and racist agendas ( Weeks, 1981). By emphasizing sex between men, there remains a risk that health promotion programmes could reinforce stereotypes of MSM as inherently ‘promiscuous’ (with all the stigma associated with multiple or anonymous sexual partners). Consequently, the marginalization experienced by affected people could be compounded. Further, the association of monkeypox with being gay could discourage MSM who do not see themselves as gay or bisexual – for example, MSM who identify as heterosexual – from adequately engaging with health information and services.

Some might argue, therefore, that it is preferable to avoid such associations between monkeypox and sex. Indeed, assertions that ‘anyone can get monkeypox’ circulate widely across health and popular outlets. However, these assertions do not reflect the data which suggest, as discussed above, that men who have sex with men have mostly contracted monkeypox in 2022, and that sexual encounters – not household contact or sharing of towels or touching door handles – have been reported as the leading route of transmission. If policy around monkeypox is embedded with narratives that fail to emphasize the role of sex between men, there is a risk that accurate, evidence-based information will not reach key groups and may lead to inadequate or inappropriate measures being implemented. Perhaps more dangerously, incomplete information about actual transmission routes and settings may lead to the assumption that gay men, based on the simple fact of being gay, are vectors of disease.

## So, is monkeypox an STI?

We want to answer this question because whether monkeypox is, or is not, an STI alters how it is understood within societies and has implications for healthcare policy, funding, and practice. On balance, we believe that monkeypox should be regarded in countries where it is not endemic as an STI because most transmissions reported to date have occurred during sexual encounters and in sexual settings. This view is limited to the current context, framed by wider assumptions about what sex, sexual health, homophobia, and public health look like. Further, we are mindful that - similar to hepatitis C ( Rauch & Wandeler, 2021) - monkeypox might be an STI only in certain communities, namely MSM, and do not discount other routes of transmission.

## From messaging and technology to social understanding and action

That monkeypox may be an STI among MSM in the current outbreak in non-endemic countries raises questions about what kind of public health messaging could be developed and delivered that both provides evidenced-based information to the communities most at risk while avoiding further stigmatization. This is further complicated by the ‘social life’ of viruses: They are always responded to within the context of pre-existing social and political agendas and ideologies, ones that often reflect prevailing power structures ( Pickersgill *et al.*, 2022; Treichler, 1987). However, co-producing communication strategies with MSM communities themselves is a vital first step.

There is a real risk that monkeypox could becomes associated with gay men through homophobic tropes. However, this risk will not be resolved by simply avoiding discussion of the epidemiological evidence. Instead, monkeypox underscores the need for concerted structural and systemic interventions that specifically address ongoing homophobia and stigma. This is particularly relevant for STIs: stigma remains a key barrier to effective prevention and care for HIV worldwide—as highlighted by UNAIDS, and it may well also determine the evolution of the current monkeypox outbreak. Responses to monkeypox could serve to propel better understandings of the intersectional inequalities that MSM experience, and accelerate the collapse of barriers to sexual health care ( Eaton *et al.*, 2015; Titanji, 2022).

Rather than focusing on systemic change, the focus of policymakers when confronted with epidemics has too often been placed on developing technological fixes: early diagnostic tests, increasing vaccine production, effective treatments, etc. As important as they are, tests, vaccines, and pharmacological treatment alone will not solve monkeypox. While an intervention may rely on a specific vaccine or drug, it requires a nuanced understanding of how communities make sense of health, disease, and risk ( Auerbach & Hoppe, 2015; Garcia-Iglesias, 2022). This is one of several vital roles that the humanities and social sciences could play in tackling monkeypox ( Pickersgill & Smith, 2021). We need to understand monkeypox not simply as an individual ailment but as a social phenomenon that exists in a context of intersecting dynamics of health and disease, equity, sexuality, and many others. It is through such understanding that we can begin to comprehend its full magnitude – and so to address it thoughtfully, carefully, and impactfully.

## Data Availability

No data are associated with this article.
