# Supplementary material for: Is mpox an STI? The societal aspects and healthcare implications of a key question
Source: Wellcome Open Res. 2023 Jul 4;7:252. Originally published 2022 Oct 11. [Version 2] doi: 10.12688/wellcomeopenres.18436.2 (PMC10329192; doi:10.12688/wellcomeopenres.18436.2)
Supplement: Peer review history

# Peer review: Reviewer response for version 2

**DOI:** 10.21956/wellcomeopenres.21801.r62031

**Published:** 2023-07-07

**Authors:** Dennis Altman

**Affiliations:** 1 La Trobe University, Melbourne, Vic, Australia

I have read the reviewed article and am totally satisfied. I have nothing further to add except to congrtaulate the writers on their contribution.

Does the article adequately reference differing views and opinions?

Partly

Are all factual statements correct, and are statements and arguments made adequately supported by citations?

Yes

Is the Open Letter written in accessible language?

Yes

Where applicable, are recommendations and next steps explained clearly for others to follow?

Yes

Is the rationale for the Open Letter provided in sufficient detail?

Yes

Reviewer Expertise:

NA

I confirm that I have read this submission and believe that I have an appropriate level of expertise to confirm that it is of an acceptable scientific standard.

# Peer review: Reviewer response for version 1

**DOI:** 10.21956/wellcomeopenres.20444.r52759

**Published:** 2022-10-24

**Authors:** Dennis Altman

**Affiliations:** 1 La Trobe University, Melbourne, Vic, Australia

This article is soundly based and deserves indexing, but it also feels already somewhat dated. The authors do not reflect on how various rich countries have responded to monkey pox, even though in many cases they have actually adopted guidelines very similar to those proposed in the letter. [I am most familiar with the Australian case, which was a good example of making information and vaccines available through services targeting MSM—while also showing an awareness of the dangers of stigmatisation.] It would be useful to examine responses in several of the countries reporting major outbreaks to ask how far they have adhered to the suggestions in this paper.

 I think it would be useful to initially provide a definition of monkey pox, and maybe a sentence or two explaining how it was first detected amongst men outside Africa. And it is not clear to me what sort of “systemic changes”—the phrase used in the final paragraph—would be required to deal with what now seems to be a declining outbreak. I am sympathetic to the call for better understanding of communities and the social dimension of epidemics, but I would like a more concrete discussion of what this would mean in terms of the current monkey pox situation.

Does the article adequately reference differing views and opinions?

Partly

Are all factual statements correct, and are statements and arguments made adequately supported by citations?

Yes

Is the Open Letter written in accessible language?

Yes

Where applicable, are recommendations and next steps explained clearly for others to follow?

Yes

Is the rationale for the Open Letter provided in sufficient detail?

Yes

Reviewer Expertise:

I have a long record of publications about HIV, but as a political scientist and not a public health expert

I confirm that I have read this submission and believe that I have an appropriate level of expertise to confirm that it is of an acceptable scientific standard, however I have significant reservations, as outlined above.

# Peer review: Reviewer response for version 1

**DOI:** 10.21956/wellcomeopenres.20444.r52760

**Published:** 2022-10-19

**Authors:** John Gilmore

**Affiliations:** 1 School of Nursing, Midwifery and Health Systems, University College Dublin, Dublin, Ireland

This letter adds significantly to contemporary discourse surrounding the WHO designated public health emergency in response to monkeypox. 

 The authors highlight important points such as the role of stigma and its impact on public health messaging and broader sexual health promotion.

 The letter stimulates thinking around the 'what if?' of categorisation of infection and disease as sexually transmitted or otherwise, this is an important consideration for us going forward in an era where much has changed in relation to prevention and treatment of STIs and indeed contemporary discourse about risk and sexual behaviour. 

 Further work on the clinical consequences around disease categorisation is warranted and no doubt the letter will stimulate clinicians to ponder this point. 

 When considering the role of stigma in health promotion we should also consider the role of community-led activism in ensuring appropriate responses to health emergencies, this is not particular to communities of gay and bisexual men but certainly evident.

 There is much to learn from the current public health emergency, and incumbent on health researchers, clinicians and organisations to take heed to the very poignant social considerations such as the ones highlighted in this letter. 

 It is commendable to see the collaboration between clinician, researcher and community activist authors.

Does the article adequately reference differing views and opinions?

Yes

Are all factual statements correct, and are statements and arguments made adequately supported by citations?

Yes

Is the Open Letter written in accessible language?

Yes

Where applicable, are recommendations and next steps explained clearly for others to follow?

Yes

Is the rationale for the Open Letter provided in sufficient detail?

Yes

Reviewer Expertise:

Inclusion Health; Gender and Sexual Minority Healthcare; Sexual Health; Nursing

I confirm that I have read this submission and believe that I have an appropriate level of expertise to confirm that it is of an acceptable scientific standard.

# Response

**Published:** 2022-10-19

**Authors:** Jaime Garcia Iglesias

**Affiliations:** The University of Edinburgh, UK

We thank Dr Gilmore for their kind feedback. We agree that this should be an ongoing conversation that speaks to deeper themes about stigma, care, and health beyond the current monkeypox outbreak. We hope, as does Dr Gilmore, that research will address these issues in the near future.
